# A longitudinal examination of the effect of physical exercise on the emotional states of college students: exploring the sense of coherence as a mediator through a cross-lagged panel analysis

**DOI:** 10.3389/fnbeh.2024.1428347

**Published:** 2024-08-22

**Authors:** Yunxia Cao, Lin Luo

**Affiliations:** ^1^School of Physical Education, Guizhou Normal University, Guiyang, China; ^2^Guizhou Vocational College of Sports, Guiyang, China

**Keywords:** physical exercise, emotional states, sense of coherence, longitudinal study, mediation analysis, college students

## Abstract

**Purpose:**

This longitudinal study aimed to investigate the causal relationship between physical exercise and emotional states among university students, focusing on the mediating role of sense of coherence.

**Method:**

A total of 1,215 university students (aged 18–25 years) were recruited and completed questionnaires assessing physical activity (International Physical Activity Questionnaire-Short Form), emotional states (Positive and Negative Affect Schedule), and sense of coherence (Sense of Coherence Scale-13) at three time points over a three-month period. Preliminary analyses included independent samples t-tests, chi-square tests, and Pearson correlations. Cross-lagged panel mediation analysis was conducted using Mplus 8.3, with bootstrapping employed to test indirect effects.

**Results:**

Results indicated that sense of coherence significantly predicted positive affect (β = 0.259–0.369, *p* < 0.001). Positive affect, in turn, predicted physical exercise (β = 0.083–0.182, *p* < 0.05), while negative affect also influenced physical exercise (β = −0.096–0.203, *p* < 0.05). Physical exercise indirectly influenced positive affect through sense of coherence (β = 0.037, *p* = 0.045), and positive affect indirectly influenced physical exercise through sense of coherence (β = 0.029, *p* = 0.028). Other indirect effects involving physical exercise, sense of coherence, and negative affect were non-significant.

**Conclusion:**

This study underscores the importance of sense of coherence in promoting emotional well-being among university students and in the reciprocal relationship between physical exercise and positive emotional states. Findings suggest that interventions targeting sense of coherence may enhance the emotional benefits of physical exercise. Future research should explore other potential mediators and moderators of the relationship between physical exercise and emotions and examine the effectiveness of sense of coherence-based interventions on well-being in this population.

## 1 Introduction

The transition to college represents a critical developmental juncture, marked by profound shifts in identity formation, social roles, and psychological maturation (Sawyer et al., 2018; Yikealo et al., 2018; Cage et al., 2021; Liu et al., 2023a). While this period offers significant opportunities for personal growth and exploration, it also presents college students with a myriad of mental health challenges (Kessler et al., 2005; Cao and Liu, 2022). Notably, Chinese college students face a unique set of cultural and societal pressures that can exacerbate these challenges.

Empirical evidence highlights a concerning rise in stress levels among Chinese college students (Cao and Liu, 2022; Liu et al., 2023a). Deeply ingrained traditional family values and parenting norms in Chinese society often place considerable pressure on students as they navigate the transition to college life and grapple with evolving social roles (Ross et al., 1999; Zhang et al., 2012; Hurst et al., 2013). Furthermore, the intensely competitive academic environment, characterized by demanding workloads and a relentless pursuit of achievement, coupled with a fiercely competitive job market, further contribute to heightened stress levels among this population (Hurst et al., 2013; Cao and Liu, 2022).

Critically, longitudinal studies focusing on Chinese college students reveal that mental well-being is not static but rather demonstrates significant fluctuation over time, with notable variations observed across different subgroups (Liu et al., 2023b,c; Xie et al., 2023; Liu et al., 2024). This underscores the importance of identifying and understanding the factors that contribute to these dynamic shifts in mental health among college students.

This study aims to investigate the impact of physical exercise on emotional states among college students, with a particular emphasis on the mediating role of Sense of Coherence (SOC). SOC, a key construct in salutogenic theory, refers to an individual’s pervasive orientation to perceive the world and their life experiences as comprehensible, manageable, and meaningful, fostering a belief in their capacity to effectively cope with life’s challenges. Emotional states encompass a wide range of affective experiences encountered in daily life, including both the reduction of negative affect (NA) and the cultivation of positive affect (PA) (Lazarus, 1991; Kahneman and Deaton, 2010). PA, in particular, has been linked to a host of cognitive and psychological benefits, including enhanced cognitive flexibility, creativity, and resilience to stress (Fredrickson, 1998, 2001).

Physical exercise, through a complex interplay of physiological and psychological mechanisms, has been shown to reduce NA, enhance PA, and consequently improve overall emotional well-being (Hamer et al., 2012; Mikkelsen et al., 2017; Kandola and Stubbs, 2020). Furthermore, emerging research suggests that physical exercise can foster the development of SOC, equipping individuals with enhanced coping mechanisms to navigate stress and adversity, ultimately promoting emotional well-being (Laudańska-Krzemińska et al., 2020; Soekmawati et al., 2022). However, there remains a dearth of longitudinal research comprehensively examining the dynamic interplay between physical exercise, emotional states, and SOC.

This study aims to address this gap by elucidating the mechanisms through which physical exercise influences emotional states among college students and by examining the mediating role of SOC in this relationship. By shedding light on these complex dynamics, this research seeks to provide a more nuanced understanding of the factors that contribute to emotional well-being among college students, ultimately informing the development of targeted interventions and support programs.

### 1.1 Relationship between PE and emotional states

Abundant studies have elucidated and sufficiently established the correlation between PE and emotional states while indicating that regular physical activity significantly benefits emotional states, especially among college students. A comprehensive body of research has evidenced that exercise is crucial for mitigating stress and anxiety in this population (Babyak et al., 2000; Ströhle, 2009; Rothon et al., 2010; Bernstein and McNally, 2017; Harvey et al., 2018). For instance, a longitudinal study by Tyson et al. (2010) found that physical activity was associated with lower levels of depression and anxiety among university students. Similarly, Feng et al. (2014) reported that regular exercise significantly reduced stress and improved mood states in Chinese college students.

PE triggers the release of endorphins and dopamine, mood-elevating neurotransmitters (Ransford, 1982; Chaouloff, 1989), boosts circulation (Numaga-Tomita et al., 2019) and oxygen supply (Lai et al., 2007), increases brain efficiency (de Sousa Fernandes et al., 2020), and reduces anxiety and tension (Mahalakshmi et al., 2020; Philippot et al., 2022). Additionally, when PE improves the physical condition and abilities of individuals, it also increases their self-esteem and confidence. These achievements are thus translated into positive emotional states, thereby fortifying affective well-being (Matta Mello Portugal et al., 2013). A study by Liu et al. (2015) demonstrated that physical activity was positively associated with self-esteem among Chinese university students, which, in turn, contributed to better emotional well-being.

PE also stimulates social interaction and teamwork, thereby offering social support and a sense of community. Engagement in group sports or team activities allows for building social connections and reducing loneliness and isolation, which, in turn, boosts emotional states (Parra-Rizo and Sanchís-Soler, 2021). Research by Zhang et al. (2012) indicated that participation in group-based exercise programs enhanced social support and reduced feelings of loneliness among college students, leading to improved emotional well-being. Finally, PE augments neuroplasticity, improving neural connections and communication, enhancing brain structure and function, and thereby increasing emotional regulation and resilience (Mahalakshmi et al., 2020).

### 1.2 Relationship between PE and SOC

Numerous studies have explored the intricate relationship between PE and SOC. SOC, delineated as individuals’ perception of congruence among their behaviors, values, and life aspirations, is pivotal in stress mitigation and disease prophylaxis. Antonovsky (1987) conceptualized that SOC encompasses the aspects of comprehensibility, manageability, and meaningfulness. Being fundamental to individual well-being, SOC captures the persistent and adaptive conviction in individuals’ capacities to efficaciously tackle internal and external environmental challenges (Frommberger et al., 1999). According to empirical research, a pronounced correlation exists between PE and SOC among individuals, revealing that those engaged in regular physical activity exhibit elevated levels of psychological well-being (Antonovsky, 1987; Hassmen et al., 2000; Edwards, 2002).

PE mainly facilitates outlining explicit objectives and action frameworks (Karamanian et al., 2020). Individuals performing PE typically need to meticulously strategize their physical training plan, in which weekly exercise frequency, duration, and intensity should be determined before and after training. Individuals’ perceptions of autonomy and self-efficacy are bolstered when these objectives are achieved (Olander et al., 2013), which then nurtures SOC. Moreover, physical activity is crucial for endorsing self-identity and self-actualization (Verkooijen and de Bruijn, 2013). Among individuals, it nurtures positive perceptions about one’s physical self-image, competencies, and health. Physical activity thus satiates appearance-and well-being-related personal anticipations, thereby magnifying identity gratification and, consequently, SOC. Furthermore, PE is related to improved emotional states and well-being. Neurotransmitters, such as endorphins, secreted during physical exertion elevate mood and well-being (You and Shin, 2017), thereby aligning positive emotional experiences with personal values and aspirations and stimulating SOC. Finally, physical activity promotes social engagement and teamwork among individuals, allowing them to develop social connections, augment social support, and cultivate a sense of belonging (Kim et al., 2021). Integrating team roles and responsibilities with individual behaviors and values further solidifies SOC (Chang et al., 2019).

In summary, using various mechanisms, including promotion of goal specificity, reinforcement of self-identity and actualization, creation of positive affective experiences, and improvement of social connectivity, PE profoundly strengthens SOC. This augmentation of perceived congruence in behaviors, values, and objectives considerably raises psychological well-being levels. Consequently, this study postulates that the degree of PE is positively correlated with SOC.

### 1.3 Mediating role of SOC in emotional states

SOC and emotional states are profoundly interconnected, and this interconnection highlights the critical role of SOC in stress mitigation, promotion of emotional equilibrium, and protection against psychological and physiological maladies. Because of SOC, individuals perceive internal stability and serenity, which then facilitates the achievement of emotional balance and positive development (Jin et al., 2022). When individuals attain congruity among behaviors, values, and objectives, they experience substantial inner peace and satisfaction, which notably diminishes negative affective experiences such as anxiety and depression, and enhances feelings of joy and fulfillment (Breslin et al., 2006; Julkunen and Ahlström, 2006). Furthermore, SOC augments self-esteem and confidence, thereby serving as a spiritual foundation for traversing life’s adversities and stressors, which then advances emotional regulation, and psychological resilience, and substantially improves emotional states (Moksnes et al., 2013; Moksnes and Espnes, 2020). Moreover, SOC promotes self-actualization and contentment, which enables individuals to achieve satisfaction by aligning their behaviors, values, and personal aims with their ideals, thereby enriching emotional experiences and contributing to overall happiness (John and Gross, 2004). SOC is also pivotal for cultivating healthy interpersonal connections by boosting social support and engendering a sense of belonging, offering emotional sustenance and security, effectively preventing negative affect, and bolstering emotional states (Gross and John, 2003; Chang et al., 2020; Fraser et al., 2021).

In summary, the extant literature suggests that PE is positively associated with emotional states and SOC, while SOC, in turn, is positively related to emotional well-being. These findings indicate that SOC may serve as a crucial mediator in the relationship between PE and emotional states among college students. By enhancing SOC, PE may not only directly contribute to improved emotional states but also indirectly foster emotional well-being through the mediating role of SOC.

**Figure 1:**
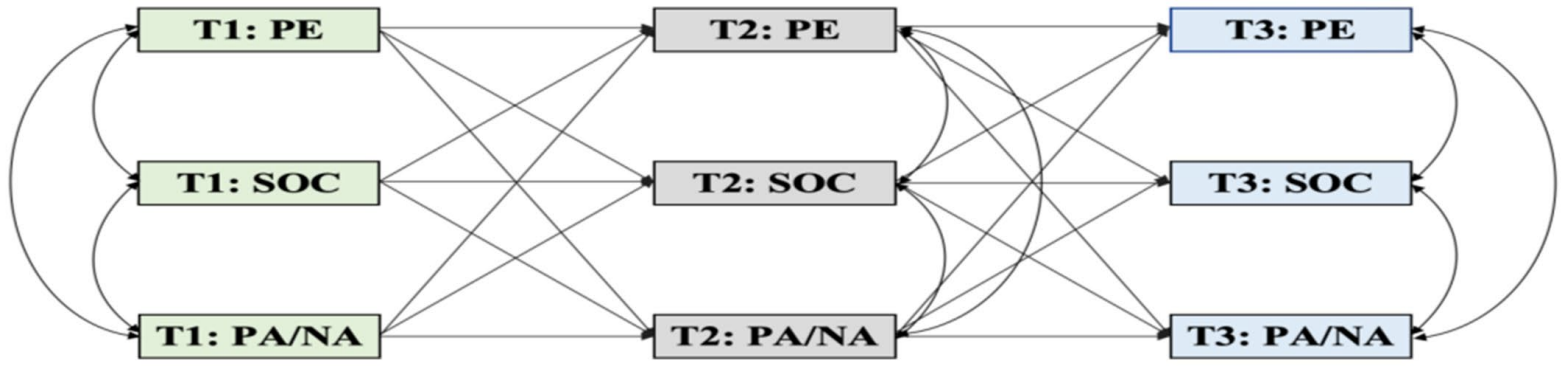
Hypothesized model. T1, Time 1 (intake); T2, Time 2 (1 month follow-up); T3, Time 3 (2-month follow-up); PE, physical exercise; SOC, sense of coherence; PA, positive affect; NA, negative affect.

To further elucidate the dynamic interplay and causal linkages among PE, SOC, and emotional states in college students, the present study employs a longitudinal, cross-lagged panel design. Based on the aforementioned theoretical and empirical foundations, we propose the following research hypotheses (Figure 1):

*H1*: Physical exercise promotes positive affect among college students.

*H2*: Sense of coherence mediates the relationship between physical exercise and positive affect among college students.

*H3*: Physical exercise reduces negative affect among college students.

*H4*: Sense of coherence mediates the relationship between physical exercise and negative affect among college students.

Figure 1 depicts the hypothesized cross-lagged panel mediation model, illustrating the direct and indirect relationships among physical exercise, sense of coherence, and emotional states (positive and negative affect) across three time points (T1, T2, and T3). The solid lines represent the hypothesized direct effects, while the dashed lines indicate the hypothesized mediating pathways via sense of coherence. The autoregressive paths [e.g., PA(T1) → PA(T2)] and cross-lagged paths [e.g., PE(T1) → SOC(T2)] are also included in the model to account for the stability and reciprocal relationships among the variables over time.

## 2 Methods

### 2.1 Participants and procedure

This longitudinal study investigated the relationship between physical exercise, emotional states, and sense of coherence among Chinese college students. Data were collected using the “Maike” digital survey platform at three time points: April, May, and June 2023. A convenience sample of undergraduate and postgraduate students from universities across China was recruited via QR codes disseminated through the WeChat platform. To maximize sample representativeness, recruitment efforts targeted students from diverse geographical locations, academic disciplines, and year levels.

#### 2.1.1 Eligibility criteria

To be eligible for inclusion in the study, participants had to be currently enrolled in an undergraduate or postgraduate program at a Chinese university, have access to a smartphone or computer with internet connectivity, and voluntarily provide informed consent. Individuals were excluded from the study if they reported any physical or mental health conditions that could potentially impede regular exercise participation or completion of the online questionnaires, regardless of whether these conditions were self-reported or medically diagnosed.

Additionally, participants were excluded if their questionnaire data were incomplete (i.e., more than 20% of items missing within any single-time point questionnaire) or if their responses exhibited potentially invalid patterns, such as straight-lining. Questionnaires flagged for potentially invalid response patterns were independently reviewed by two researchers, and exclusion decisions were made via consensus based on pre-established criteria to ensure objectivity. Finally, participants who did not provide informed consent were excluded from all study procedures.

Prior to initiating any study procedures, all potential participants received comprehensive information about the study and provided informed consent. The questionnaire surveys were administered in three phases, spaced 1 month apart, to allow for the examination of changes over time. This study received ethical approval from the Guizhou Normal University Institutional Review Board (approval number: 202300005) and was conducted in accordance with the ethical principles outlined in the latest Declaration of Helsinki.

#### 2.1.2 Sample attrition and bias examination

Initially, 1,215 student participants were enrolled in the study. However, due to attrition, the sample size decreased to 974 and 904 participants at the second and third data collection points, respectively. Complete data across all three time points were available for 533 students, who comprised the final analytic sample.

To assess potential attrition bias, independent samples t-tests were conducted to compare the initial participant pool (*n* = 1,215) with the final analytic sample (*n* = 533) on key demographic variables and study variables (physical exercise levels, emotional states, and sense of coherence). The absence of statistically significant differences between these groups suggested that data missingness was random and unlikely to have introduced substantial bias into the study findings. Table 1 provides a detailed overview of the demographic characteristics of the final analytic sample (*n* = 533).

**Table 1:** Internal consistency testing of study variables (*n* = 533).

Scales	Items	Cronbach’s alpha		
		T1	T2	T3
PANAS	20	0.808	0.876	0.875
Positive affect (PA)	10	0.851	0.903	0.903
Negative affect (NA)	10	0.887	0.905	0.899

T1, Time 1 (intake); T2, Time 2 (one-month follow-up); T3, Time 3 (2-month follow-up); PA and NA belongs to PANAS.

### 2.2 Measures

#### 2.2.1 Physical exercise

Physical exercise levels were quantified using the Physical Activity Rating Scale-3 (PARS-3) developed by Liang (1994). The PARS-3 consists of three items assessing the intensity, duration, and frequency of physical exercise, each rated on a 5-point Likert scale (1–5). An exercise volume score is calculated using the following formula: Volume = Intensity * Time * Frequency, resulting in a total score ranging from 0 to 100. Based on their scores, students were categorized into three physical activity level groups: low (≤19 points), moderate (20–42 points), and high (≥43 points). The PARS-3 has demonstrated good reliability and validity in Chinese college student samples, with a Cronbach’s α of 0.78 and test–retest reliability of 0.82 (Liang, 1994; Wang et al., 2020). Example items include: “How intense is your physical activity in general?,” “How long do you usually exercise each time?,” and “On average, how many times a week do you engage in physical exercise?”

#### 2.2.2 Sense of coherence

Participants’ sense of coherence was assessed using the 13-item Sense of Coherence scale (SOC-13), adapted to Chinese by Bao et al. (2006). The SOC-13 employs a 7-point Likert scale to measure three dimensions: comprehensibility, manageability, and meaningfulness. Higher total scores indicate a stronger sense of coherence. The Chinese version of the SOC-13 has shown good psychometric properties, with Cronbach’s α ranging from 0.76 to 0.91 and test–retest reliability of 0.78 (Bao et al., 2006; Zhang et al., 2011). Example items include: “When you face difficult situations, do you feel you are able to find a way to handle them?” and “Do you have the feeling that your life has a purpose and meaning?”

#### 2.2.3 Emotional states

Positive and negative emotional states were evaluated using the Chinese version of the Positive and Negative Affect Schedule (PANAS), developed by Huang et al. (2003). This scale comprises two 10-item subscales, each rated on a 5-point Likert scale (1 = very slightly or not at all, 5 = extremely), measuring the intensity of positive and negative emotional experiences, respectively. Higher scores on each subscale indicate greater intensity of the corresponding emotional experience. While overall emotional state was considered, positive and negative affect were also analyzed as relatively independent constructs. The Chinese version of the PANAS has demonstrated good reliability and validity, with Cronbach’s α of 0.85 for the positive affect subscale and 0.83 for the negative affect subscale (Huang et al., 2003; Li et al., 2015). Example items include: Positive affect—“Excited, interested, strong, enthusiastic, alert”; Negative affect—“Distressed, upset, scared, nervous, hostile.”

### 2.3 Statistical analysis

Data analysis for this study was conducted in three primary stages. First, to ensure the data met the assumptions of multivariate normality, a Doornik-Hansen test was performed in Stata 16.1, examining the key variables of age, physical exercise, sense of coherence, positive affect, and negative affect. The significance level for this test was set at *p* < 0.05, with only data meeting this criterion considered to fulfill the assumption of multivariate normality and suitable for further analysis.

Next, preliminary analyses were conducted using SPSS 26.0 to gain a comprehensive understanding of the sample characteristics and relationships between variables. Specifically, independent samples *t*-tests and chi-square tests were employed to compare participants included and excluded from the study on key variables, ensuring sample representativeness. Descriptive statistics, including means, standard deviations, and frequencies, were calculated and analyzed. Pearson correlation coefficients were calculated to examine the bivariate associations between study variables. Cronbach’s α coefficients were also computed to assess the reliability of the scales, with values greater than 0.7 considered acceptable for further analysis. To facilitate interpretation, Pearson correlation coefficients were categorized into five levels based on Cohen’s (2013) criteria: negligible (≤0.19), low (0.20–0.39), moderate (0.40–0.59), moderate-high (0.60–0.79), and high (≥0.80).

Finally, to examine the hypothesized causal relationships and mediating effects among the variables, a cross-lagged panel mediation model was analyzed using Mplus 8.3. Path coefficients, representing the magnitude and direction of effects between variables in the model, were estimated. Total, indirect, and direct effects were also calculated to quantify the influence of variables on each other. A bias-corrected bootstrapping procedure with 5,000 resamples was employed to test the significance of the indirect (mediating) effects. A 95% confidence interval that did not include zero was considered to indicate a statistically significant mediating effect.

## 3 Results

### 3.1 Test of common method biases

When the singular survey method was used for data collection, a potential spurious covariance was introduced among the variables. Therefore, the Harman single-factor test was applied to the initial dataset (*N* = 1,215) to evaluate common method biases (Livingstone et al., 1997). The foremost principal component only contributed to 20.749% of the total variance from the 36 extracted components without rotation, which was well beneath the 40% threshold. This result confirms that common method biases were not a significant problem in this study. In later survey rounds, adjustments were also made in the questionnaire item sequence as a precautionary measure.

### 3.2 Descriptive analysis

The sample consisted of 533 university students (average age: 19.38 ± 1.22 years) from different Chinese provinces, and 50.28% of these participants were female students. The dropout rate was 56.13%. Table 2 presents the demographic details of participants and the outcomes of the dropout analysis. Chi-square tests conducted to examine ethnic background and economic status unveiled no statistically significant differences between the groups included and excluded from the study, evidenced by *p* values for ethnicity (χ^2^ = 1.958, *p* = 0.162) and economic status (χ^2^ = 0.812, *p* = 0.666). Furthermore, independent samples t-tests conducted on SOC, emotional states, and PE levels at T1 revealed no statistically significant differences for PA (*t* = −0.050, *p* = 0.960), NA (*t* = 0.148, *p* = 0.883), and PE (*t* = 1.847, *p* = 0.065). However, a significant difference was found in SOC at T1 between the included and excluded groups (*t* = 2.031, *p* = 0.043), with the excluded group having a slightly higher SOC score (54.69 ± 9.11) than the included group (53.67 ± 8.04). These findings suggest that participant attrition was largely random, with the exception of a small difference in baseline SOC scores, which should be considered when interpreting the study’s conclusions.

**Table 2:** Demographic characteristics of the participants (*N* = 1,215).

Variable		Included data (*n* = 533)	Excluded data (*n* = 682)	*x*^2^/*t*	*p*-value
		Mean ± SD or *n* (%)			
Sex	Male	265 (49.72)	408 (59.82)	12.365	<0.001
	Female	268 (50.28)	274 (40.18)		
Age		19.38 ± 1.22	20.10 ± 1.78	8.066	<0.001
Ethnicity	Han	296 (55.53)	406 (59.53)	1.958	0.162
	Minorities	237 (44.47)	276 (40.47)		
Household	City	126 (23.64)	203 (29.77)	6.939	0.031
	Town	102 (19.14)	136 (19.94)		
	Rural	305 (57.22)	343 (50.29)		
Grade Level	Freshman	482 (90.43)	413 (60.56)	139.775	<0.001
	Sophomore	28 (5.25)	132 (19.35)		
	Junior	17 (3.19)	82 (12.02)		
	Senior	0	18 (2.64)		
	Graduate	6 (1.13)	37 (5.43)		
Major	Sociology	270 (50.66)	271 (39.74)	51.390	<0.001
	Science	132 (24.77)	175 (25.66)		
	Engineering	113 (21.20)	131 (19.21)		
	Art and Sport	18 (3.37)	105 (15.39)		
Economic conditions	Low	212 (39.77)	267 (39.15)	0.812	0.666
	Intermediate	318 (59.66)	408 (59.83)		
	High	3 (0.57)	7 (1.02)		
SOC(T1)		53.67 ± 8.04	54.69 ± 9.11	2.031	0.043
SOC(T2)		54.37 ± 7.29			
SOC(T3)		53.00 ± 6.52			
PA(T1)		30.05 ± 5.53	30.03 ± 6.08	−0.050	0.960
PA(T2)		28.90 ± 6.66			
PA(T3)		29.62 ± 6.01			
NA(T1)		24.11 ± 6.17	24.16 ± 6.20	0.148	0.883
NA(T2)		23.13 ± 6.68			
NA(T3)		25.29 ± 6.01			
PE(T1)		21.89 ± 19.11	24.03 ± 20.78	1.847	0.065
PE(T2)		20.76 ± 18.56			
PE(T3)		19.27 ± 16.34			

T1, Time 1 (intake); T2, Time 2 (one-month follow-up); T3, Time 3 (2-month follow-up); SD, standard deviation; SOC, sense of coherence; PA, positive affect; NA, negative affect; PE, physical exercise.

Key variables were evaluated here at three-time intervals: PA, NA, SOC, and PE (Table 3). A comparative analysis across these time points revealed notable statistical differences in PA, NA, and SOC among the groups at each measurement stage. Nonetheless, PE exhibited no statistically significant differences among the groups (*F* = 2.834, *p* = 0.059).

**Table 3:** The comparison of baseline data for the study variables across different periods (*n* = 533).

Variable	T1	T2	T3	*F*	*p-*value
	Mean ± SD				
PE	21.90 ± 19.11	20.76 ± 18.56	19.27 ± 16.34	2.834	0.059
SOC	53.67 ± 8.04	54.37 ± 7.29	53.00 ± 6.52	4.730	0.009
PA	30.05 ± 5.53	28.89 ± 6.66	29.62 ± 6.01	4.907	0.008
NA	24.11 ± 6.17	23.13 ± 6.68	25.29 ± 6.01	15.735	<0.001

T1, Time 1 (intake); T2, Time 2 (one-month follow-up); T3, Time 3 (2-month follow-up); SD, standard deviation; SOC, sense of coherence; PA, positive affect; NA, negative affect; PA and NA belongs to PANAS; PE, physical exercise.

As shown in Table 4, the most frequently reported exercise intensity at baseline (T1) was “small strength, not too nervous movements” (EI-2, 31.89%), followed by “intense and sustained exercise of moderate intensity” (EI-3, 30.96%). Follow-up assessments at 1 month (T2) and 2 months (T3) revealed a similar pattern, suggesting that the distribution of exercise intensity remained relatively stable over the study period.

**Table 4:** Frequency distribution of exercise intensity (*n* = 533).

Exercise intensity (EI)	T1		T2		T3	
	Frequency	Percent (%)	Frequency	Percent (%)	Frequency	Percent (%)
EI-1	86	16.14	92	17.26	85	15.95
EI-2	170	31.89	168	31.52	184	34.52
EI-3	165	30.96	172	32.27	170	31.89
EI-4	95	17.82	86	16.14	81	15.20
EI-5	17	3.19	15	2.81	13	2.44

T1, Time 1 (intake); T2, Time 2 (1 month follow-up); T3, Time 3 (2-month follow-up); EI-1, Light exercise; EI-2, Small strength not too nervous movements; EI-3, Intense and sustained exercise of moderate intensity; EI-4, Shortness of breath, sweating a lot of great strength, but not lasting movement; EI-5, Shortness of breath, sweating a lot of great intensity of lasting movement.

### 3.3 Bivariate analysis

**Figure 2:**
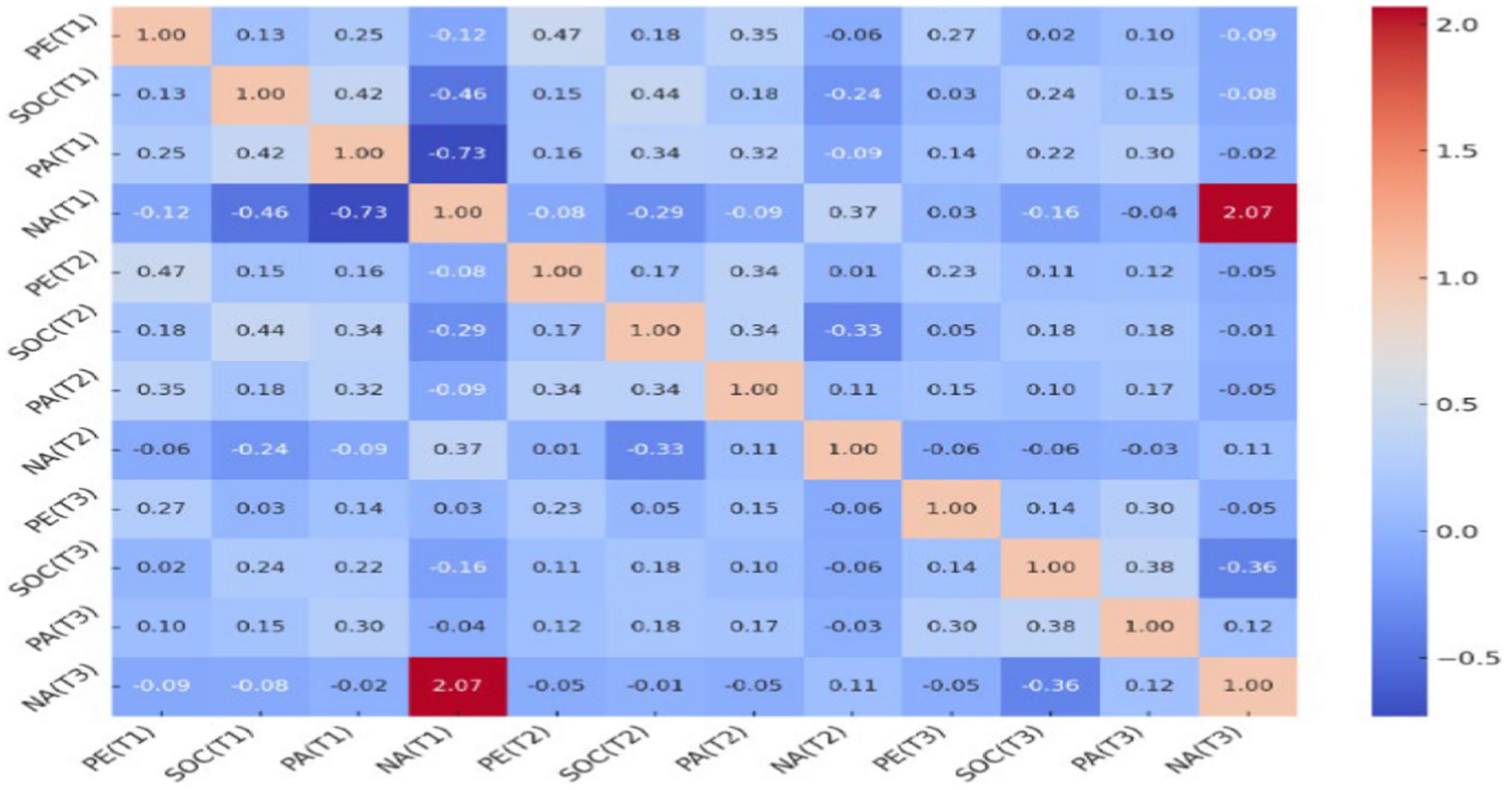
Heatmap of the correlation matrix. The correlation coefficients among various variables are shown, with the intensity of the color reflecting the strength and direction of the correlation. Positive correlations are depicted in warmer colors, while negative correlations are indicated in cooler colors.

To investigate the relationships between PE, PA, NA, and SOC over three-time points, Pearson correlation coefficients were calculated. PE at Time 1 [PE(T1)] and SOC at Time 2 [SOC(T2)] were significantly correlated (*r* = 0.177, *p* < 0.001). Similarly, PE(T1) and PA at Time 2 [PA(T2)] were significantly correlated (*r* = 0.346, *p* < 0.001). Furthermore, SOC at Time 1 [SOC(T1)] and PA at Time 2 [PA(T2)] were significantly related (*r* = 0.179, *p* < 0.001), whereas SOC(T1) and NA at Time 2 [NA(T2)] exhibited a significant negative correlation (*r* = −0.237, *p* < 0.001). Additional correlation findings are detailed in Figure 2.

### 3.4 Path analysis of PE and emotional states

In this study, cross-lagged models were used to rigorously explore the dynamics between PE, PA, NA, and SOC across three temporal intervals (T1, T2, and T3), thereby concentrating specifically on their influence on emotional states. Based on preliminary correlational findings, a cross-lagged mediation model was constructed to evaluate the interactive association between PE and emotional states—comprising both PA and NA—while demographic factors such as gender, place of residence, and economic status were integrated as control variables into the model so as to mitigate potential confounding influences.

**Figure 3:**
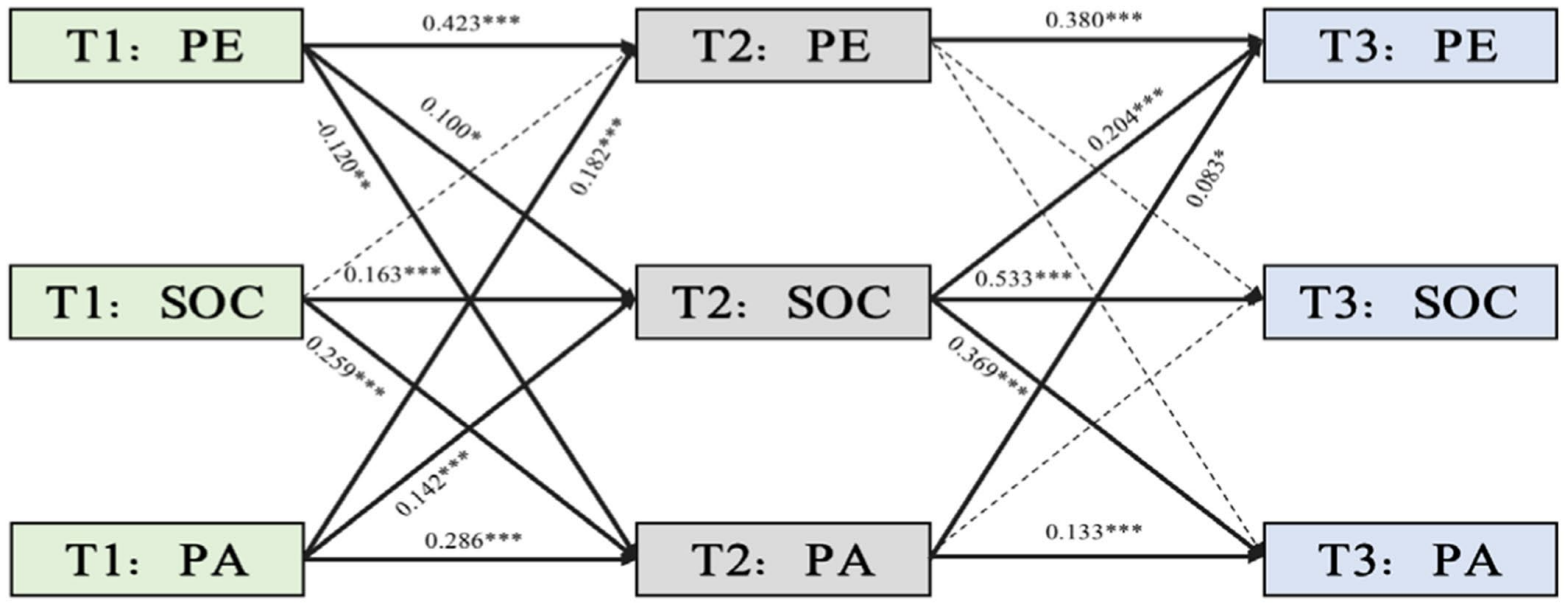
A standardized cross-lagged path model among PE, SOC, and PA. T1, Time 1 (intake); T2, Time 2 (one-month follow-up); T3, Time 3 (2-month follow-up); SOC, sense of coherence; PA, positive affect; PE, physical exercise. Solid lines indicate significance, and dotted lines signify non-significance. The residuals are not shown; *p < 0.05, **p < 0.01, ***p < 0.001.

Autoregressive coefficients for emotional state-related variables exhibited statistically significant continuity across the study phases. The path analysis (Figure 3) revealed that from T1 to T2 and T2 to T3, SOC exerted a significant predictive effect on PA, thereby highlighting the substantial causal impact of SOC on emotional states over time (T1 → T2: β = 0.259, *p* < 0.001; T2 → T3: β = 0.369, *p* < 0.001). Concurrently, the model demonstrated that PA made a statistically significant contribution to PE across both intervals (T1 → T2: β = 0.182, *p* < 0.001; T2 → T3: β = 0.083, *p* = 0.032), whereas the predictive capacity of SOC on PE was not statistically significant. This finding indicated that PA sustains a unidirectional predictive relationship with PE, and SOC alone impacts emotional states, delineating the meticulous interplay among physical activity, SOC, and the spectrum of affective experiences.

**Figure 4:**
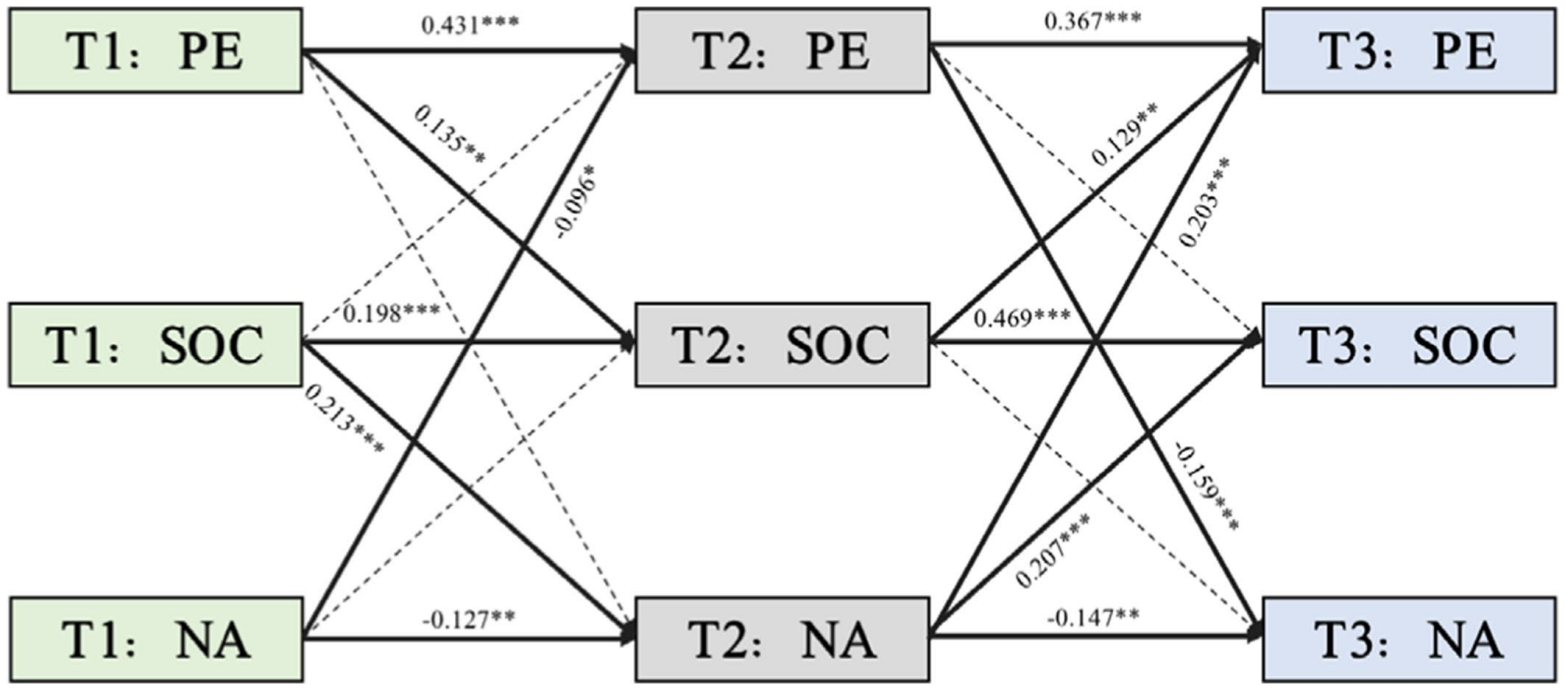
A standardized cross-lagged path model among PE, SOC, and NA. T1, Time 1 (intake); T2, Time 2 (one-month follow-up); T3, Time 3 (2-month follow-up); SOC, sense of coherence; NA, negative effect; PE, physical exercise. Solid lines indicate significance, dotted lines indicate non-significance. The residuals are not shown; *p < 0.05, **p < 0.01, ***p < 0.001.

Moreover, the path analysis further elucidated (Figure 4) that NA significantly influenced PE across both T1 to T2 and T2 to T3 intervals (T1 → T2: β = −0.096, *p* = 0.022; T2 → T3: β = 0.203, *p* < 0.001). Yet, the predictive capacity of SOC on both PE and NA was not statistically significant (SOC(T1) → PE(T2): β = 0.035, *p* = 0.358; SOC(T1) → NA(T2): β = 0.213, *p* < 0.001; SOC(T2) → PE(T3): β = 0.129, *p* = 0.002; SOC(T2) → NA(T3): β = 0.016, *p* = 0.723), which revealed a unidirectional causal relationship between NA to PE. This accentuates that the components of emotional states and PE are intricately related over time. These insights imply that while SOC significantly influences the positive dimensions of emotional states, it seems to exert more constrained direct effects on PE and NA, which highlights the complex interplay among various aspects of emotional states and physical activity.

The subsequent analysis (Table 5) uncovered that path coefficients for the mediating role of SOC in the PE–PA relationship were statistically significant, which indicates meaningful indirect effects (PE(T1) → SOC(T2) → PA(T3): β = 0.037, 95% CI [0.001, 0.073], *p* = 0.045). Path coefficients highlighting the mediating role of SOC in the PA–PE dynamic relationship were also statistically significant (PA(T1) → SOC(T2) → PE(T3): β = 0.029, 95% CI [0.003, 0.055], *p* = 0.028). Conversely, path coefficients concerning the SOC-mediated influence of PE on NA through SOC and the SOC-mediated reciprocal influence of NA on PE were not statistically significant (PE(T1) → SOC(T2) → NA(T3): β = 0.002, 95% CI [−0.014, 0.018], *p* = 0.791; NA(T1) → SOC(T2) → PE(T3): β < 0.001, 95% CI [−0.013, 0.012], *p* = 0.961). This result thus underscores the differential mediating effects of SOC between the positive and negative dimensions of emotional states and PE.

**Table 5:** Standardized path analysis among variables at different time points.

PA path	Coefficient (β)	S.E.	*p*-value	95%CI
PE(T1) → PE(T2)	0.423	0.036	<0.001	0.354 ~ 0.493
PE(T1) → SOC(T2)	0.100	0.043	0.020	0.016 ~ 0.184
PE(T1) → PA(T2)	−0.120	0.040	0.003	−0.200 ~ −0.041
SOC(T1) → PE(T2)	0.002	0.039	0.960	−0.074 ~ 0.078
SOC(T1) → SOC(T2)	0.163	0.043	<0.001	0.079 ~ 0.246
SOC(T1) → PA(T2)	0.259	0.039	<0.001	0.181 ~ 0.336
PA(T1) → PE (T2)	0.182	0.040	<0.001	0.104 ~ 0.026
PA(T1) → SOC(T2)	0.142	0.044	0.001	0.055 ~ 0.229
PA(T1) → PA(T2)	0.286	0.041	<0.001	0.206 ~ 0.366
PE(T2) → PE(T3)	0.380	0.036	<0.001	0.309 ~ 0.450
PE(T2) → SOC(T3)	0.017	0.036	0.629	−0.053 ~ 0.088
PE(T2) → PA(T3)	0.046	0.040	0.242	−0.031 ~ 0.124
SOC(T2) → PE(T3)	0.204	0.038	<0.001	0.129 ~ 0.278
SOC(T2) → SOC(T3)	0.553	0.030	<0.001	0.494 ~ 0.612
SOC(T2) → PA(T3)	0.369	0.037	<0.001	0.297 ~ 0.442
PA(T2) → PE(T3)	0.083	0.039	0.032	0.007 ~ 0.159
PA(T2) → SOC(T3)	−0.015	0.036	0.684	−0.086 ~ 0.056
PA(T2) → PA(T3)	0.133	0.040	0.001	0.055 ~ 0.210
PE(T1) → SOC(T2) → PA(T3)	0.037	0.018	0.045	0.001 ~ 0.073
PA(T1) → SOC(T2) → PE(T3)	0.029	0.013	0.028	0.003 ~ 0.055
**NA path**	**Coefficient (β)**	**S.E.**	***p*-value**	**95%CI**
PE(T1) → PE(T2)	0.431	0.038	<0.001	0.356 ~ 0.506
PE(T1) → SOC(T2)	0.135	0.046	0.003	0.045 ~ 0.224
PE(T1) → NA(T2)	0.078	0.045	0.086	−0.011 ~ 0.166
SOC(T1) → PE(T2)	0.035	0.038	0.358	−0.040 ~ 0.110
SOC(T1) → SOC(T2)	0.198	0.042	<0.001	0.117 ~ 0.280
SOC(T1) → NA(T2)	0.213	0.041	<0.001	0.133 ~ 0.294
NA(T1) → PE (T2)	−0.096	0.042	0.022	−0.178 ~ −0.014
NA(T1) → SOC(T2)	−0.002	0.046	0.958	−0.093 ~ 0.089
NA(T1) → NA(T2)	−0.127	0.046	0.005	−0.217 ~ −0.038
PE(T2) → PE(T3)	0.367	0.036	<0.001	0.297 ~ 0.437
PE(T2) → SOC(T3)	−0.003	0.035	0.931	−0.072 ~ 0.066
PE(T2) → NA(T3)	−0.159	0.042	<0.001	−0.241 ~ −0.076
SOC(T2) → PE(T3)	0.129	0.041	0.002	0.048 ~ 0.210
SOC(T2) → SOC(T3)	0.469	0.035	<0.001	0.401 ~ 0.538
SOC(T2) → NA(T3)	0.016	0.046	0.723	−0.074 ~ 0.107
NA(T2) → PE(T3)	0.203	0.041	<0.001	0.122 ~ 0.283
NA(T2) → SOC(T3)	0.207	0.038	<0.001	0.132 ~ 0.281
NA(T2) → NA(T3)	−0.147	0.046	0.001	−0.237 ~ −0.057
PE(T1) → SOC(T2) → NA(T3)	0.002	0.008	0.791	−0.014 ~ 0.018
NA(T1) → SOC(T2) → PE(T3)	<0.001	0.006	0.961	−0.013 ~ 0.012

T1, Time 1 (intake); T2, Time 2 (one-month follow-up); T3, Time 3 (2-month follow-up); SOC, sense of coherence; PA, positive affect; NA, negative affect; PE, physical exercise.

To further test the complete mediation effect of SOC, we compared the fit indices of the hypothesized model with alternative models that included direct paths from PE(T1) to PA(T3) and from PA(T1) to PE(T3). The results showed that adding these direct paths did not significantly improve the model fit [Δχ^2^(2) = 3.641, *p* = 0.162], suggesting that SOC fully mediated the relationships between PE and PA over time.

## 4 Discussion

This study delves into the interplay of emotional states, physical exercise (PE) levels, and sense of coherence (SOC) among university students.

### 4.1 Results discussion

A negative association between negative affect (NA) and PE levels was observed from the first time point (T1) to the second (T2), aligning with previous research suggesting that reducing negative emotions may enhance individuals’ willingness to engage in physical activity (Modolo et al., 2011; Liu X. et al., 2023). Interestingly, this trend reversed from T2 to T3, with increased negative affect corresponding to higher PE levels. This reversal might reflect the structured physical education courses prevalent in Chinese universities, particularly for non-sports majors. Integrated into academic evaluations, these courses aim to promote physical well-being and cultivate lasting exercise habits, potentially serving as a stress-relief mechanism toward the semester’s end (Baghurst and Kelley, 2014). This finding also aligns with some studies suggesting that, under specific circumstances, negative emotions can motivate physical exercise (Eklund and Crawford, 1994). However, the expected mediating role of SOC on the relationship between PE levels and NA was not observed. Further exploration of the conditions under which SOC might influence this dynamic relationship is warranted.

Furthermore, our findings confirmed the consistent, positive influence of positive affect (PA) on PE levels across the observation period, corroborating a direct causal relationship between positive emotions and PE levels. This aligns with previous findings demonstrating that positive emotional states can significantly motivate individuals to participate in physical activity (Helfer et al., 2015).

This study also highlighted the significant mediating role of SOC in the relationship between PE levels and PA, underscoring the importance of SOC for emotional well-being among university students. Higher SOC levels were significantly associated with higher PA, consistent with research identifying SOC as a determinant of positive affect (Oztekin and Tezer, 2009). However, no predictive relationship of PE levels on SOC was found, similar to a longitudinal study on community-dwelling older adults where SOC did not significantly influence physical activity levels (Kukihara et al., 2018). This suggests that the relationship between PE levels and SOC is complex and influenced by factors not covered in this study, such as the nature of exercise, individual perceptions of coherence, and other mediating variables. Future research should explore these factors to better understand the conditions under which PE levels might impact SOC.

### 4.2 Research significance

This study revealed a crucial mediating role of SOC in the relationship between PA and PE levels, indicating that SOC is essential for enhancing the positive impact of PE levels on emotional states. This finding expands on previous research by highlighting the importance of considering SOC when examining the emotional benefits of PE for university students. This suggests that interventions aimed at enhancing SOC among university students could be significantly beneficial to their mental and physical well-being by fostering self-esteem and confidence, fulfilling needs for self-actualization, and providing social support (Posadzki et al., 2010).

By investigating the mechanisms through which PE levels influence emotional well-being among university students, this longitudinal study provides empirical evidence for addressing emotional health challenges within this population. The findings have important theoretical and practical implications. Theoretically, this study contributes to the existing literature by providing a more nuanced understanding of the dynamic relationships between PE, SOC, and emotional states among university students. Practically, the findings suggest that interventions aimed at enhancing SOC and promoting PE could be effective strategies for improving emotional well-being among university students. Universities should consider incorporating such interventions into their student health and well-being programs.

### 4.3 Limitations and future directions

While this study offers valuable insights into the intricate relationships between PE levels, emotional states, and SOC among university students using a cross-lagged model, it has limitations.

Participants were primarily recruited from a single university, limiting the generalizability of the findings. Future research should recruit a more diverse sample from multiple universities to enhance the representativeness of the findings. The study relied on self-reported measures, which are susceptible to potential biases, including recall inaccuracies, social desirability effects, and response bias, potentially compromising the objectivity and accuracy of the collected data. Incorporating objective measurement techniques, along with other data collection techniques like direct behavioral observation and physiological assessments, could provide a more comprehensive and accurate portrayal of the studied phenomena. The study’s limited time span might have masked long-term dynamic relationships and influences between PE levels, emotional states, and SOC. The two-month follow-up period might be insufficient to capture the full range of changes in these variables over time. Future studies should employ longitudinal designs with longer timeframes to explore the enduring effects of these variables over time more thoroughly. Furthermore, future research must explore the role of cultural and social contexts in shaping the interplay between PE levels, emotional states, and SOC. Valuable insights can be gained regarding how cultural norms, societal expectations, and educational practices influence these variables and their interrelationships when investigating these relationships across diverse cultural backgrounds.

Despite these limitations, this study significantly contributes to our understanding of the dynamic relationships between PE, emotional states, and SOC among university students. Addressing the identified limitations and incorporating the suggested methodological refinements in future research can significantly enrich the field, providing more profound, actionable insights and practical implications for promoting emotional and physical well-being among individuals in university settings and beyond.

## 5 Conclusion

This study illuminates the significant predictive power of a sense of coherence on positive emotional states, highlighting its crucial role in emotional well-being. Furthermore, the study reveals a positive feedback loop between positive emotional states and physical exercise. These findings suggest that interventions such as mindfulness training, stress management workshops, and social support programs could enhance students’ sense of coherence, thereby maximizing the positive effects of physical exercise on emotional states. The age of artificial intelligence presents opportunities to leverage these technologies to develop personalized mindfulness training applications, intelligent stress monitoring tools, and social platforms. These resources can provide more precise and effective support and interventions for university students, ultimately improving their sense of coherence and emotional well-being.

## Data availability statement

The raw data supporting the conclusions of this article will be made available by the authors, without undue reservation.

## Ethics statement

The studies involving humans were approved by The Ethics Committee of Guizhou Normal University, China (Approval No.: 202300005). The studies were conducted in accordance with the local legislation and institutional requirements. The participants provided their written informed consent to participate in this study.

## Author contributions

YC: Conceptualization, Data curation, Formal analysis, Investigation, Methodology, Project administration, Resources, Software, Validation, Visualization, Writing – original draft. LL: Conceptualization, Funding acquisition, Methodology, Supervision, Writing – original draft, Writing – review & editing.
